# Intertwined Relation between the Endoplasmic Reticulum and Mitochondria in Ischemic Stroke

**DOI:** 10.1155/2022/3335887

**Published:** 2022-04-28

**Authors:** Jianhua Peng, Dipritu Ghosh, Jinwei Pang, Lifang Zhang, Shigang Yin, Yong Jiang

**Affiliations:** ^1^Department of Neurosurgery, The Affiliated Hospital of Southwest Medical University, Luzhou 646000, China; ^2^Laboratory of Neurological Diseases and Brain Function, The Affiliated Hospital of Southwest Medical University, Luzhou 646000, China; ^3^Institute of Epigenetics and Brain Science, Southwest Medical University, Luzhou 646000, China; ^4^Academician (Expert) Workstation of Sichuan Province, The Affiliated Hospital of Southwest Medical University, Luzhou 646000, China; ^5^Sichuan Clinical Research Center for Neurosurgery, The Affiliated Hospital of Southwest Medical University, Luzhou 646000, China

## Abstract

In ischemic stroke (IS), accumulation of the misfolded proteins in the endoplasmic reticulum (ER) and mitochondria-induced oxidative stress (OS) has been identified as the indispensable inducers of secondary brain injury. With the increasing recognition of an association between ER stress and OS with ischemic stroke and with the improved understanding of the underlying molecular mechanism, novel targets for drug therapy and new strategies for therapeutic interventions are surfacing. This review discusses the molecular mechanism underlying ER stress and OS response as both causes and consequences of ischemic stroke. We also summarize the latest advances in understanding the importance of ER stress and OS in the pathogenesis of ischemic stroke and discuss potential strategies and clinical trials explicitly aiming to restore mitochondria and ER dynamics after IS.

## 1. Introduction

Stroke is defined as the sudden loss of neurological function because of a vascular accident and is the leading cause of death and disability worldwide [1]. The underlying molecular mechanism in ischemic and hemorrhagic stroke has been extensively studied. However, morbidity and mortality associated with both conditions remain high. Ischemic stroke (IS) directly results from disruption of blood circulation to the brain and makes up approximately 87% of all known cases of stroke [1]. Strategies aiming to reduce damage and disability associated with ischemic stroke focus on regulating endogenous protective mechanisms, thus minimizing the postischemic stroke insults. Those novel approaches are the regulation of endoplasmic reticulum (ER) stress, oxidative stress (OS), and antioxidant treatment in postischemic stroke conditions.

The endoplasmic reticulum (ER) is widely distributed within neuronal dendrites, dendritic spines, axons, presynaptic nerve terminals, and growth cones and is an essential cellular organelle for secreted and membrane protein folding [2]. Disruption of this standard mechanism of ER induces a pathological state known as ER stress [3]. ER stress triggers an adaptive response called unfolded protein response (UPR), which initially leads to inhibition of protein synthesis followed by later upregulation of protein folding genes and disposal of misfolded proteins [3]. Transcriptional and translation aspects of the UPR protect neurons from being overwhelmed by misfolded ER proteins; however, if the disruption period is prolonged, the UPR aims towards apoptosis. Unlike other cells in the human body, postmitotic neuronal cells are highly susceptible to ER stress because of the loss of replicating power and primarily depend on UPR for its survival. Similarly, glial cells have homogeneous susceptibility towards ER stress because of their highly developed secretory pathways.

On the other hand, OS directly results from excitotoxicity in cerebrovascular accidents. The release of the neurotransmitter glutamate has been identified as the primary culprit behind excitotoxicity. Under the hypoperfusive state, there is a diminished level of oxygen and glucose, which elevates glutamate release, causing overexcitation of postsynaptic neurons. Glutamate excitotoxicity has also been associated with mitochondrial dysfunction, generation of reactive oxygen species (ROS), reactive nitrogen species (RNS), disruption of calcium homeostasis, and loss of mitochondrial membrane potential. Severe mitochondrial damage can further elicit increased levels of ROS and eventually lead to apoptosis.

This review summarizes the correlation between ER stress and OS, secondary brain damage as a direct result of ER stress and OS, potential target therapies, and future advancement in this field.

## 2. Pathophysiology of ER Stress and Oxidative Stress after Ischemic Stroke

### 2.1. Oxidative Stress after Ischemic Stroke

**Figure 1:**
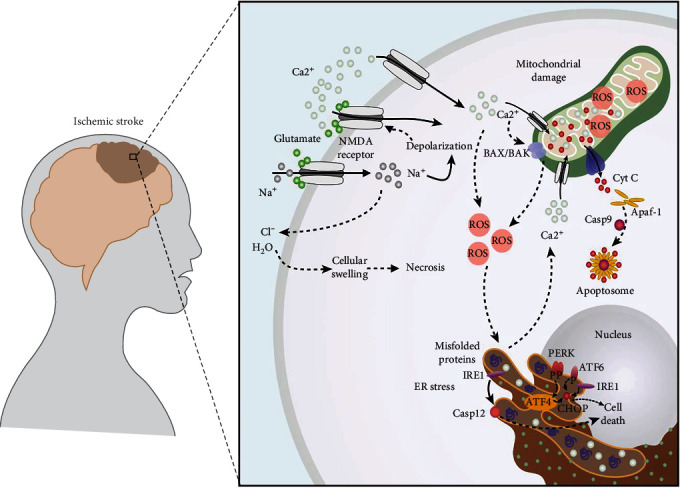
Pathological signaling pathway involving mitochondrial damage, ROS production, and ER stress. Postischemic accident produces glutamate excitotoxicity, leading to increased influx of Ca^2+^ ions. The excessive influx of Ca^2+^ causes mitochondrial damage and an increase in ROS production, which further causes ER stress. Prolonged ER stress causes activation of unfolded protein response through protein kinase RNA-like endoplasmic reticulum kinase (PERK), activating transcription factor 6 (ATF6), and inositol-requiring kinase 1*α* (IRE1*α*). Activated PERK phosphorylates eukaryotic factor 2a (elF2*α*), which suppresses global protein synthesis and activates transcription factor 4 (ATF-4). PERK activation and subsequent elF2*α* phosphorylation elevate ATF4 which induces cell death by upregulation of Bcl-2 family members and the key transcription factor C/EBP homologous protein (CHOP), which in turn regulates transcription of Bcl-2 family members. Thus, CHOP plays a crucial role in determining the cellular fate by alternating the levels of Bcl-2 members towards apoptosis. Under the ischemic condition, neuronal apoptosis is instigated by Bcl-2 family members, including Bax and Bak, which cause mitochondrial structural damage and enable the release of cytochrome C. Release cytochrome C interacts with apoptotic protease activating factor-1 (Apaf-1) to form the apoptosome, and caspase-9 is activated. Caspase-12, an ER membrane-associated caspase, is upregulated by glutamate excitotoxicity, which facilitates caspase cascade, further provoking cellular death.

OS is considered a principal factor of brain injury in cerebrovascular accidents. Excessive ROS production poststroke is the main culprit behind OS. Therefore, stroke leads to the peroxidation of lipids, proteins, and nucleic acids, leading to mitochondrial dysfunction and DNA damage, which subsequently induces cell death (Figure 1).

#### 2.1.1. The Mechanism Involved in Oxidative Stress after Ischemic Stroke

OS is defined as an imbalance between oxidants and antioxidants in favor of the oxidants, leading to a disruption of redox signaling and control and molecular damage [4]. Despite being relatively smaller than other vital organs in the body, the human brain consumes 20% of the total basal oxygen (O2) to support adenosine triphosphate (ATP) intensive neuronal activity and is highly susceptible to ischemia. OS has been identified as one of the foremost causes of brain injury after IS. Transient or permanent disruption of cerebral blood flow is typical of IS and thereby causes brain tissue injury and even death. People might argue that restoring blood supply to the ischemic region might reduce the ischemic insults.

However, studies showed that restoring the blood supply to the ischemic region might further escalate the ischemic injury due to many ROS molecules produced during this process. This paradoxical phenomenon is called reperfusion injury [5]. ROS molecules play a crucial role in producing OS-related neuronal damage after IS. There are three main types of ROS molecules produced during an ischemic insult, namely, superoxide anion (O2−), hydroxyl radical (OH−), and hydrogen peroxide (H2O2). After cerebral ischemia, the associated brain damage is caused by the excessive amount of ROS through (1) interfering with and inhibition of protein synthesis, along with DNA damage; (2) mitochondrial structural damage, impairing electron transport chain and reducing ATP production; (3) lipid peroxidation (LPO) of the unsaturated fatty acids in the cell membrane; and (4) disruption of the blood-brain barrier (BBB).

Under physiological conditions, ROS production is highly scrutinized by the antioxidant system. However, ischemic insults disrupt the equilibrium in favor of ROS production. The human body comprises of two antioxidant systems, namely, the enzymatic antioxidant system, which include superoxide dismutase (SOD), glutathione peroxidase (GPX), glutathione, and catalase, and the nonenzymatic antioxidant system, including glutathione, melatonin, carotenoids, vitamin C, and vitamin D. Thus, stroke-induced OS has mainly been associated with excess ROS production, and use of antioxidant therapy is advocated in clinical practice.

#### 2.1.2. Mitochondrial Damage

Mitochondrial damage is an essential pathological event during the early stages of IS [6]. Mitochondria are the primary source of postischemic stroke ROS generation. The excessive ROS production by mitochondria after stroke attenuates the electron transport chain, decreases ATP production, and subsequently damages mitochondrial structure and function. Studies have been undertaken to determine the involvement of OS and mitochondrial dysfunction in stroke and found that postischemia reduction in ROS production protects against OS. Furthermore, mitochondrial ROS production or mitochondrial OS removal can provide a favorable outcome in postischemic stroke conditions.

Protein kinase A (PKA)/cAMP-response element-binding protein (CREB) and 12/15-lipooxygenase (12/15-LOX) have been identified as regulators of mitochondrial ROS production. Xue and colleagues reported that activation of PKA triggers CREB phosphorylation, preserves mitochondrial function, and minimizes ROS production in ischemic conditions, thereby protecting the cerebral cortical neurons from OS [7]. Similarly, inhibition of the 12/15-LOX pathway has also been identified as beneficial in ischemic conditions [8, 9]. Thus, it is crucial to maintain mitochondrial ROS production to achieve a favorable prognosis in postischemic conditions.

### 2.2. ER Stress and Response

Under the ischemic condition, neuronal apoptosis is instigated by proapoptotic genes, namely, Bcl-2 family members including Bcl-2-associated X protein (Bax) and Bcl-2 homologous antagonist killer (Bak), which cause mitochondrial structural damage and enable the release of cytochrome C. Release cytochrome C interacts with apoptotic protease activating factor-1 (Apaf-1) to form the apoptosome, and caspase-9 is activated [10]. Activation of caspase-9 further creates downstream activation of the caspase-3, which cleaves poly [ADP-ribose] polymerase 1 (PARP-1), leading to DNA damage. Overactivation of PARP-1 harms ATP and nicotinamide adenine dinucleotide (NADH) production, which translates into energy failure and cellular necrosis [11]. Similarly, histone methylation of p53 has been closely associated with apoptosis in postischemic stroke, and demethylation of the methylated histone is catalyzed by Jumonji domain-containing proteins (JMJD) family. Among them, JMJD3 has been more associated with Bax and caspase-3 and is expressed throughout the brain, including neurons [12].

ER acts as the primary site for the processing and folding newly synthesized proteins and plays a critical role in calcium (Ca^2+^) storage and signaling. Depleting ER Ca^2+^ and OS can trigger impairment of ER function and activate UPR. Under chronic ER stress, UPR evokes cellular apoptosis [13]. Thus, ER stress plays a critical role after IS.

#### 2.2.1. Unfolded Protein Response

The UPR mainly serves to restore ER function by inhibiting protein synthesis, disposal of misfolded proteins, and later upregulation of protein folding genes. The activation of UPR response is triggered by protein kinase RNA-like endoplasmic reticulum kinase (PERK), activating transcription factor 6 (ATF6), and inositol-requiring kinase 1*α* (IRE1*α*). PERK, ATF6, and IRE1*α* under the physiological condition in neurons interact with Grp78. ER dysfunction results in phosphorylation of PERK and IRE1*α* and cleavage of ATF6 (P90) to ATF6 (P50) [14]. Activated PERK phosphorylates eukaryotic factor 2a (elF2*α*), which suppresses global protein synthesis and activates transcription factor 4 (ATF-4).

Under prolonged ER stress, PERK activation and subsequent elF2*α* phosphorylation elevate ATF4, inducing cell death by upregulation of Bcl-2 family members and the critical transcription factor C/EBP homologous protein (CHOP), which in turn regulates transcription of Bcl-2 family members. Wang et al. reported that PERK-mediated p-eIF2*α* suppresses protein synthesis in the postischemic brain, neuroprotective in experimental stroke. Thus, PERK activation or elF2*α* phosphorylation plays a crucial role in determining the cellular fate by alternating the levels of apoptosis after IS. Furthermore, caspase-12, an ER membrane-associated caspase, is upregulated by glutamate excitotoxicity, which facilitates caspase cascade, further provoking cellular death.

Munoz and colleagues identified that PERK is a crucial regulator of mitochondrial function and morphology during ER stress [15]. Similarly, Ca^2+^ and glutamate have also been an intertwined relation in ER stress and mitochondrial dysfunction in ischemic conditions [16]. Therefore, we can determine the central regulators of OS and their correlation with mitochondrial dysfunction and ER stress.

#### 2.2.2. ER Stress Link to Inflammation and BBB Disruption

In the central nervous system, the close link between ER stress and inflammation is likely to contribute to the integration of metabolic homeostasis and ER function. Inflammatory response limits tissue damage and facilitates tissue repair under ER stress. However, prolonged stress will directly link inflammation with cell death. After ischemic events, UPR seeks to inform cells in disordered homeostasis through inflammatory pathways, which is very important to reduce the innate immune response to ER stress [17]. ER stress determines cell fate through several previously reported inflammatory response-related pathways, including toll-like receptors (TLR) signaling pathway, nuclear factor *κ*B (NF-*κ*B) signaling pathway, Jun N-terminal kinase/activator protein 1 (JNK/AP1) signaling pathway, and mitogen-activated protein kinase (MAPK) signaling pathway [18].

As a vital organelle for protein secretion and modification, disruption of proper ER function leads to the impairment of UPR and leads to the abnormality of cell structure and function. Ischemic conditions trigger ER stress, which triggers UPR and ER to enter a pathological state with Ca^2+^ equilibrium loss. In contrast, Ca^2+^ overload in endothelial cells has been identified as the main factor of cerebral swelling and BBB disruption [19]. Cerebral edema and BBB disruption occur quickly after IS, causing intracellular swelling, and subsequently, cerebral edema enters the stage of ionic and vasogenic edema. Thus, effective treatment of ER stress is particularly crucial for BBB integrity and cerebral edema.

## 3. Pharmacological Therapies

Current clinical treatment of IS primarily focuses on the acute and subacute stages of the injury process. The critical stage of the disease is defined as the time frame within 4.5 hours from the appearance of the first symptoms. However, current guidelines on thrombolytic [20] and endovascular [21] treatment are limited to various factors such as economic level or uneven understanding of stroke, and the proportion of patients who can receive thrombolysis or endovascular therapy within 4.5 or 6 hours is still deficient. On the other hand, patients receiving thrombolysis or mechanical thrombectomy within the said window of time still may suffer from secondary brain injury [22]. Therefore, finding and developing new therapeutic targets demise secondary brain injuries (Table 1).

### 3.1. Mitochondria as a Potential Target

Mitochondria are the main source of postischemic stroke intracellular ROS generation. Changes in ROS levels affect the expression or activity of proteins associated with mitochondrial dynamics, which in turn affects mitochondrial fusion and division [23]. Mitochondrial fission divides mitochondrion into two smaller mitochondria and is regulated by dynamin-1-like protein (Drp1). Mitochondrial fission conventionally occurs at the ER-mitochondrial contact site. During ischemic conditions, an increase in ROS level promotes Drp1 activation through phosphorylation of Ser616 [24]. Interestingly, Drp1 serine 637 phosphorylation inhibits mitochondrial fission. Phosphorylation of Drp1 at tyrosine 266, 368, and 449 leads to mitochondrial division and neuronal death [25]. Zhang and colleagues demonstrated that mitochondrial division increases the production of ROS. However, inhibition of mitochondrial division can restore ROS levels to normal [26]. Mitochondrial division is Drp1-dependent, and knock out (KO) of Drp1 reduces OS-induced mitochondrial fragmentation [27]. Interestingly, inhibition of Drp1 also reduces the oligomerization of Bax and apoptotic factors after IS, thereby reducing cerebral infarction volume [28].

#### 3.1.1. Treatment Targeting Mitochondria after Ischemic Stroke

Mitochondrial dynamics after IS are closely linked to failure in energy metabolism, ROS, apoptosis, and autophagy. Therefore, the molecular mechanisms associated with mitochondrial dynamics are essential to be targeted to produce a favorable prognosis in postischemic stroke patients.

Zhou and colleagues demonstrated that atractylenolide III and AG490 (inhibitor of JAK2) therapy in middle cerebral artery occlusion (MCAO) mice reduces Drp1 phosphorylation (p-Drp1), translocation, and mitochondrial division through Janus kinase 2/signal transducer and activator of transcription 3 (JAK2/STAT3) pathway, thereby attenuating cerebral edema and neurological deficits [29]. Like Drp1, OPA1 (OPA1 mitochondrial dynamin-like GTPase) is essential for mitochondrial fusion, reducing infarct size, inhibition of neuronal death, and reducing cerebral reperfusion stress through the Yap-Hippo pathway [30]. Under cobalt chloride-induced hypoxia in the mouse hippocampal cell culture, a decrease in Opa1 and p-Drp1 levels was observed. Treatment with 4-chloro-N-(naphthalen-1-ylmethyl)-5-(3-piperazin-1-ylphenoxy) thiophene-2-sulfonamide (B355252) restores levels of Opa1 and p-Drp1, preserves mitochondrial stability, restores mitochondrial membrane potential, and reduces ROS production [31].

Other potential treatment options include miR-7a-5p injections after cerebral ischemia, which can subdue *α*-synuclein. *α*-Synuclein promotes mitochondrial fragmentation, OS, autophagy, and promote neuronal death. Thus, subduing *α*-synuclein levels with miR-7a-5p injections after IS can be beneficial [32]. In a mouse model of bilateral common carotid artery occlusion, subcutaneous injection of granulocyte colony-stimulating factor (G-CSF) could reduce autophagy marker Beclin-1 and apoptosis-related proteins, such as Bax, Bak, and Drp1. However, G-CSF also promotes mitochondrial fusion protein, Opa1. Consequently, G-CSF maintains mitochondrial dynamics by reducing apoptosis and protecting neurons in cerebral ischemia [33]. Nitric oxide synthase 3 (NOS3) inhibitor regulates mitochondrial Rho GTPase2 levels, thus promoting axon functional recovery.

However, the clinical application of these potential target therapies is subjected to further in-depth studies assessment in terms of efficacy, toxicity studies, and clinical trials. Thus, a potential target therapy answering all the underlying postischemia disruption can be expected in the near future.

### 3.2. ER as a Targeted Treatment

Autophagy is defined as the process of degradation of worn-out proteins, damaged organelles, and misfolded proteins through a lysosome-dependent regulation mechanism to maintain cellular homeostasis. As discussed earlier, ischemic conditions trigger ER stress, which triggers UPR and ER to enter a pathological state with Ca^2+^ equilibrium loss. This signifies ER stress in tight association with autophagy within the central nervous system. The ER stress is associated with autophagy through three signaling pathways, namely, PERK, ATF6, and IRE1*α* [34]. Therefore, regulating these key pathways may provide an expected outcome by minimizing cellular loss in postischemic conditions.

3-Methyladenine (3-MA) is a phosphatidylinositol 3-kinases (PI3K) inhibitor. PI3K controls mTOR activation, a key regulator of autophagy [35]. However, 3-MA is showcased to aggravate cerebral ischemia-induced ER stress and increase activated proapoptotic caspase-12 and caspase-3 protein levels in vivo and vitro [36]. Interestingly, it reduced the expression of autophagy genes and autophagy and UPS co-regulatory genes. The positive outcome of 3-MA can be attenuated by using an autophagy inducer, rapamycin, during hypoxia-reoxygenation-induced brain injury [37].

ER stress-induced by tunicamycin and thapsigargin has been documented to protect against ischemic brain injuries [38]. Preischemic stroke melatonin treatment was reported to reduce acute neuronal injuries through inhibiting ER stress-dependent autophagy via PERK and IRE1 signaling pathways [39]. However, other research showcases the complexity of crosstalk between ER stress and autophagy in IS. Neither reduction of ER stress nor enhancing autophagy have a neuroprotective effect in neurons under ischemic conditions [40]. Because of the discrepancy and unknown molecular mechanism between ER stress and autophagy, in-depth studies need to be conducted. Nevertheless, ER stress remains a critical factor in IS.

#### 3.2.1. Treatment Options for Endoplasmic Reticulum Stress

Hairy and enhancer of split 1 (Hes1) have been identified as a regulator of ER stress. A study by Li and colleagues showed that knocking down Hes1 aggravates IS in the temporary middle cerebral artery occlusion model by ER stress-dependent apoptosis via PERK/elF2*α*/ATF4/CHOP signaling pathway [41]. Similarly, Homer1a, a short scaffold protein overexpression, was illustrated to preserve mitochondrial function by regulating cytochrome c release, less ROS production, reduced ATP and mitochondrial membrane potential loss, decreased caspase-9 activation, and inhibition of ER stress by inhibiting the PERK pathway [42]. Therefore, Hes1 and Homer1a might be potential target treatment opportunities.

Over the years, microRNA (miR) studies have established themselves as a crucial factor in postischemic stroke conditions as target treatments. miR-9-5p has been shown to attenuate IS by targeting endoplasmic reticulum metallopeptidase 1 (ERMP1), thus minimizing ER stress in the MCAO rat model [43]. Similarly, upregulation of miR-216a provides neuroprotection against ischemic injury by negative regulation of JAK2/STAT3 [44]. Dong and colleagues exemplify the role of miR-7 in ER stress through HERPUD2 [45]. However, like other forms of target treatments, the data gathered does not proceed with their use in clinical practice.

Posiphen is a stereoisomer of the acetylcholinesterase inhibitor phenserine, and it has implicated its use in mild cognitive impairment and Alzheimer's disease [46]. Recently, studies have shown the beneficial role of Posiphen in the reduction of ER stress [47], and combined therapy with pifithrin-*α* enhances neurogenesis and functional recovery after IS [48]. Although Posiphen has substantial evidence implicating potential use in the early phase of postischemic stroke in favor of the desired prognosis, yet, the studies fail to imply the prognostic value of Posiphen use in the late stage of postischemic stroke.

Celecoxib is a well-known anti-inflammatory, recently proclaimed to reduce ER stress by decreasing the expression of glucose-related protein 78 (GRP78), CHOP, and caspase-12 after 48 hours of reperfusion. Additionally, celecoxib was also showcased to enhance the IRE1-UPR pathway, further reducing ER stress [49].

Several other potential targets can be listed to reduce ER stress after and before the IS. Still, for this review, we focus on the possible target treatment that satisfies our target pathways and keeps the review simple for the reader's digestion.

### 3.3. Reactive Oxygen Species

After an intracerebral ischemic accident, the most sensible treatment option is to restore the blood supply in the ischemic region. However, by doing so, the patients are subjected to a paradoxical phenomenon known as ischemic reperfusion injury (IRI). It has been postulated that IRI is closely associated with an increase in OS by upregulating ROS production. Although ROS plays an essential role in intracellular signaling and immune response, cellular antioxidants cannot scavenge redundant ROS production, creating many pathological responses and ultimately cellular damage [50]. To minimize IRI, the administration of antioxidant reagents for scavenging ROS has been widely applied.

#### 3.3.1. Antioxidant Treatment after Ischemic Stroke

Antioxidant treatment to regulate global ROS production is a viable strategy for treating IS. After a cerebrovascular accident, cyclooxygenases and mitochondria generate ROS due to their enzymatic activity; however, ROS generation is the principal function of the NADPH oxidase (NOXs) family NOX2 and NOX4 activity which is the major contributor of OS following a cerebrovascular accident [51].

Inhibiting NOXs may be an efficient strategy for minimizing ROS-related cellular damage. Apocynin, a naturally occurring NOX inhibitor, was showcased to decrease infarct volume by reducing the level of apoptosis and inhibiting OS. However, the therapeutic window for apocynin is narrow [52]. Other known NOX inhibitors include Gp91ds-tat and ebselen; however, their use postischemia is highly scrutinized because of the low oral bioavailability, associated side effects, and nonspecific nature [53, 54]. Even with substantial drawbacks, a placebo-controlled, double-blinded clinical trial was conducted on 300 patients diagnosed with acute IS (< 48 hours) to determine the efficacy of ebselen. The trial concluded with the verdict that the use of ebselen 300 mg/d, administered within 24 hours, produces a favorable outcome in patients with IS [55]. Even though with clear indications of a favorable outcome and little to no side effects, ebselen remains debatable. NADPH oxidase inhibitors are promising treatment options but are need to be subjected to further experimental studies.

With recent development in the mode of delivery of drugs, nanomedicine has gained popularity in producing specific ROS scavenger effects with a promising prognosis. The efficacy of nanomedicines is highly associated with the types of material the delivery vessel is made of. t-PA@iRNP is a thrombolytic and antioxidant nanomedicine that encapsulates tissue plasminogen activator (t-PA) in conjugation with 4-amino-2,2,6,6-tetramethylpiperidine-1-oxyl (4-amino-TEMPO), size of ∼50 nm, and pH acidic trigger of 6.2. Administration of t-PA@iRNP provides dual benefits of thrombolytic activity and a significant decrease in ROS production in the MCAO mice model. Furthermore, the antioxidant effect of 4-amino-TEMPO efficiently avoids subarachnoid hemorrhage induced by t-PA, providing potential dual therapy via synergic effect [56, 57]. Polyoxometalate (POM) nanoclusters are also novel bioresponsive nanomaterial containing molybdenum ions (Mo5+ and Mo6+). Intrathecal administration of POM in rats reduces IRI-induced OS, apoptosis, edema, and infarct volume of the brain up to ∼50% [58]. The practical and precise use of nanomedicine can provide a new avenue for future treatment options.

## 4. Clinical Studies

Clinical studies or clinical trials are prospective biomedical research studies on human patients to determine the dosage, safety, and efficacy of a new potential treatment. Over the years, several trials were conducted to determine effective treatment options for IS. However, because of the complicated and intertwined pathophysiological mechanism of IS, an effective treatment option is yet to be discovered.

As mentioned earlier, G-CSF maintains mitochondrial dynamics by reducing apoptosis and protecting neurons in cerebral ischemia [33]. However, phase *ΙΙ* trial on forty-nine acute IS patients administered with 150 *μ*g/body/day and 300 *μ*g/body/day of G-CSF within 24 hours of the onset of symptoms did not show functional recovery or reduction in infarct volume at 3 months after onset, compared to the placebo group [58]. The investigators suspect that the lack of effectiveness of G-CSF is due to the small sample size and plans to conduct further trials as combination therapy with t-PA. However, with a long-standing history of G-CSF trials, no clinical trial data published to date were able to show significant triumphant effects of G-CSF treatment in a large cohort of IS patients.

Glutamate, a neurotransmitter, has been identified as the main culprit behind excitotoxicity. Glutamate excitotoxicity has also been associated with mitochondrial dysfunction, ROS generation, RNS, calcium homeostasis disruption, and mitochondrial membrane potential loss. One of the suggested methods of attenuating glutamate excitotoxicity is blocking the N-methyl-D-aspartate (NMDA) receptor. Caffeinol (a combination of a low dose of caffeine and ethanol) was demonstrated to have an anti-ischemic property through the NMDA antagomir effect [59]. A clinical trial carried on twenty IS patients, treated with a combination of caffeinol (caffeine 8-9 mg/kg + ethanol 0.4 g/kg intravenously, started by 4 hours after symptom onset), hypothermia, and t-PA, suggest feasible treatment approach with no adverse effects towards caffeinol [60]. However, the trial failed to demonstrate the prognostic effect of the said combination treatment compared to placebo. It mainly focused on the feasibility and tolerability, thus, creating concern regarding the necessity of the said complex approach.

ROS scavenger compounds have powerful implications in postischemic stroke conditions to minimize the OS and prevent secondary brain damage. One such promising scavenger compound is edaravone, a member of the substituted 2-pyrazolin-5-one class [61]. A newer form, edaravone dexborneol, produced a more favorable functional outcome among female patients with acute IS in a multicenter, randomized, double-blind, comparative, phase III clinical trial compared to edaravone when administered within 48 hours of the onset of symptoms [62]. Xu and colleagues also demonstrated dose-depended functional recovery of the edaravone dexborneol group compared to the edaravone group with a higher modified Rankin score (mRSscore) in medium-dose (37.5 mg by 30-min intravenous infusion every 12 hours, for 14 consecutive days) group [63].

Recently, the role of iron as a target to prevent stroke-induced ROS-depended neurodegeneration was put under the spotlight due to new emerging evidence showing that regulation of ferroptosis in the ischemic brain parenchyma is protective in experimental IS [64, 65]. A double-blinded, randomized, placebo-controlled, dose-finding phase II clinical trial of intravenous deferoxamine along with t-PA in acute IS patients (*n* = 62) was carried out to evaluate the safety, tolerability, and therapeutic efficacy of iron chelator deferoxamine mesylate (DFO). Participants were randomly divided into placebo, 20 mg/kg/day DFO, 40 mg/kg/day DFO, and 60 mg/kg/day DFO group with primary t-PA treatment. Continuous placebo administration and all DFO arms were initiated during t-PA infusion and carried out for 72 hours. Iron saturation was determined using blood transferrin levels. A positive trend to efficacy was observed in moderate-severe IS patients (NIHSS >7) receiving 40-60 mg/kg/day DFO with 50-58% good outcome at 90 days compared to 31% in the placebo group [66].

While discussing some of the previous clinical studies, it is appropriate to talk about some of the propitious ongoing studies as well. Various multifactorial aspects of IS are still under examination. For instance, a multicenter, prospective, randomized, open-label, blinded end-point (PROBE) trial is being carried out to determine the efficacy of exenatide in acute IS patients. Exenatide is a glucagon-like peptide-1 receptor (GLP1R) agonist, widely used as a medication to treat diabetes mellitus type 2. Postischemic stroke hyperglycemia occurs in 50% of the patients, reducing thrombolysis efficacy, increasing the risk of hemorrhage, and increasing infarct size, translating into a poor prognosis in patients. The proposed mechanism of action of exenatide in acute IS conditions is to reduce OS, inflammation, and edema. The primary outcome of the study is to measure the improvement in the neurological outcome of patients administered with exenatide within 9 hours of the onset of symptoms (5 *μ*g subcutaneously twice daily for five days) along with standard treatment protocols (NCT03287076).

As mentioned earlier, overactivation of PARP-1 due to caspase cascade results in energy failure and cellular death [13], and inhibition of PARP-1 can be beneficial. JPI-289 is a PARP-1 inhibitor, which was shown to reduce infarct volume and improve neurological function and neuroprotection in the MCAO animal study model [67]. The positive outcome in the animal study model has led to a multicenter, randomized, double-blind, placebo-controlled, phase IIa clinical trial to evaluate the efficacy and safety of JPI-289 in acute IS patients. The primary aim of the study is to determine the infarct growth ratio from the initial presentation (4 days from the initial presentation). Patients will be administered with JPI-289 within 6.5 hours from the initial symptom development along with standard treatment protocols (NCT03062397).

The complexity of the previous NMDA receptor inhibition approach using a combination of caffeinol, hypothermia, and t-PA is not feasible under clinical settings because of the highly sophisticated nature of the procedure with constant monitoring and also maybe due to failure in obtaining consensus from the patient's family. 2-Hydroxy-5-(2,3,5,6-tetrafluoro-4-trifluoromethyl-benzylamino)-benzoic acid (Neu2000) is a derivate of aspirin and sulfasalazine, showcased to prevent both NMDA neurotoxicity and OS with a single bolus in the MCAO animal study model [68]. However, the safety and efficacy of Neu2000 in human IS patients have not been explored. A phase II, double-blind, randomized, placebo-controlled, multicenter study was undertaken in South Korea (2016) to determine the safety and efficacy of Neu2000; however, the study has not published the trial results so far (NCT02831088) (Table 2).

The Inconsistency in the outcome of the treatment options is due to the challenge of enrolling a sufficient number of representative patients with comparable characteristics and differences in patient demographics. The difference in demographics translates into a difference in genetic and habitual behavior. So, to determine a dose-dependent multidemographic prognostic value of a potential drug, large multicenter, randomized, double-blinded cohort studies need to be carried out.

## 5. Prospective and Conclusion

In this review, we have summarized new advancements in recent years concerning the mechanisms of brain ischemia, ER stress, and OS. As outlined above, UPR, mitochondrial damage, and ROS production are the main focus of ischemic redox research. It is clear that ER stress, OS, and ROS exert significant effects in the process of secondary cerebral ischemic damage, and the difference in severity determines the prognostic outcome in the postischemic stroke conditions. What is interesting is the fact that ER stress and OS are a double-edged sword. Therefore, re-establishing cellular homeostasis after an ischemic incident needs to be carried out with caution.

Perhaps the answer to ischemic redox stress may lie within the epigenetic aspect of the research. IS is a complex, multifactorial disease in which a wide plethora of pathological processes are simultaneously set in motion, and modulation of a single molecular factor is unlikely to be sufficient to attenuate or reverse the progression of stroke pathology. Epigenetic alterations such as DNA methylation, histone modifications, and RNA modifications are potent modulators of gene regulation. An accumulating body of evidence suggests that they play a pivotal role in regulating brain remodeling after stroke [69–71]. Further efforts are being made to understand the underlying epigenetic regulation in IS [72, 73]. Because of the complexity of IS, we speculate that epigenetics research is still at its inception point, and prolonged, in-depth studies are required before we find an answer to IS.

On the other hand, the nanomedicine drug delivery system is a promising treatment option, especially t-PA@iRNP. In the hyperacute stage of IS, administration of t-PA is advocated for qualified patients in clinical practice because of the sentimental nature of the drug [74]. Various combination therapy with t-PA have been suggested over the years, but none like t-PA@iRNP. If t-PA@iRNP can prove its proclaimed prognostic effect in clinical trials [56], there is a chance of potentially safer treatment option in the hyperacute stage of IS.

Remote ischemic conditioning (RIC) is a process by which cycles of temporary ischemia, typically through a manual or electronic tourniquet, applies to a limb above systolic blood pressure, which confers systemic protection against future ischemic attack injuries in remote vascular territories. RIC is popularized as a viable treatment approach for IS in recent years. RIC is an experimental medical procedure that aims to boost the body's natural protection mechanism against tissue injury under ischemic conditions to reduce the severity of the ischemic injury. Although RIC has been shown to reduce infarct size and improve functional outcomes in the experimental animal models [75], it is still an experimental procedure in human beings, and large-scale trials are necessary to determine the true benefits. Many clinical studies are being carried out in different countries to evaluate the viability of RIC in the different ischemic populations (NCT03868007, NCT03740971, NCT03481777, and NCT03375762). We speculate that these ongoing clinical studies will provide sufficient knowledge to establish the viability of RIC in IS.

In conclusion, IS is a multifactorial, multilevel complex disease involving a plethora of ROS and ER stress processes. The treatment method for such a disease cannot be one-dimensional. Future research studies need to understand the multifactorial nature of IS and the feasibility of treatment options in clinical settings.

## Figures and Tables

**Table 1:** Summarize table of potential drugs and their targets, species studied on, study model, and mechanism of action. Six.

Name	Target	Species	Model	Mechanism of action	Reference
Hes1	ER	C57 mice	tMCAO	PERK/elF2*α*/ATF4/CHOP	[38]
Homer1a	ER	C57BL/6 mouse primary neurons	OGD	PERK	[39]
miR-9-5p	ER	SD rats	MCAO	ERMP1	[40]
miR-216a	ER	SD mice	MCAO	JAK2/STAT3	[41]
miR-7	ER	C57BL/6 mouse primary neurons	OGD	HERPUD2	[42]
Posiphen	ER	SD rats/C57BL/6 mice	MCAO	NMDA receptor/APP + p53	[44, 45]
Celecoxib	ER	SD rats	tMCAO	GRP78/CHOP/Caspase-12	[46]
Atractylenolide III and AG490	Mitochondria	C57BL/6 mice	MCAO	JAK2/STAT3	[26]
B355252	Mitochondria	HT22 cells	Hypoxia	Opa1 and p-Drp1	[28]
miR-7a-5p	Mitochondria	SHR and *α*-Syn−/− mice	MCAO	*α*-Synuclein	[29]
G-CSF	Mitochondria	Swiss Webster mice	BCAO	p-Akt	[30]
Apocynin	ROS	SD rats	MCAO	NADPH oxidase	[49]
Gp91ds-tat	ROS	COS-22 and COS-Nox2 cells	Superoxide	NADPH oxidase	[50]
Ebselen	ROS	Human	AIS patients	NADPH oxidase	[52]
t-PA@iRNP	ROS	ICR mice	MCAO	Thrombolytic and antioxidant	[53]
POM	ROS	SD rats	I/R	ROS scavenger	[54]

APP: amyloid precursor protein; BCAO: bilateral common carotid artery occlusion; I/R: ischemia/reperfusion; OGD/R: oxygen glucose deprivation/re-oxygenation; P-Akt: Akt phosphorylation; SHR: spontaneously hypertensive rats.

**Table 2:** Summarize table of ongoing and concluded clinical studies with agents, trial number, countries participating, the proposed mechanism of action, intervention time, and their outcome of the study.

Agent	Trial no.	Country	Duration	Proposed mechanism	Intervention time	Status	Final verdict
G-CSF	UMIN000006607	Japan	Dec. 2011-Mar. 2015	Maintains mitochondrial dynamics	Within 24 h; infusion twice a day for 5 days	Completed	No functional recovery or reduction in infarct volume at 3 months
Caffeinol + hypothermia	NCT00299416	USA	Feb. 2003-Aug. 2009	NMDA antagomir	Within 4 h; along with t-PA	Completed	Feasibility and tolerability
Edaravone dexborneol	NCT02430350	China	May 2015-Dec. 2016	ROS scavenger	Within 48 h; one dose every 12 h for 14 days	Completed	Dose-dependent function recovery; maximum in medium-dose group (37.5 mg)
Deferoxamine mesylate (DFO)	NCT00777140	Spain	Sep. 2008-Dec. 2011	Reduce iron-dependent ROS production	Within 3 h; t-PA intervention followed by DFO intravenous perfusion for 72 h	Completed	Positive efficacy trend in moderate-severe ischemic stroke patients (NIHSS >7) at 90 days
Neu2000	NCT02831088	South Korea	July 2016-Present	NMDA antagonist and antioxidant	Within 8 h; 9 consecutive infusions of Neu2000 at 12 h interval	Completed	—
Exenatide	NCT03287076	Australia	Jan. 2017-present	Reduce OS, inflammation, and edema by GLP1R agonist	Within 9 h; along with standard treatment protocols	Ongoing	—
JPI-289	NCT03062397	South Korea	Dec. 2016-present	Reduce mitochondrial dysfunction	Within 24 h; along with standard treatment protocols	Ongoing	—
Remote ischemic conditioning (RIC)	NCT03868007	China	Mar. 2019-present	Antioxidative, anti-inflammatory, and mitochondria protective effect	Within 4 h, twice daily for 14 days	Ongoing	—
	NCT03740971	China	Dec. 2018-present		Within 48 h, twice daily	Ongoing	—
	NCT03481777	Denmark	Apr. 2018-present		After 6 h, twice daily for 7 days	Ongoing	—
	NCT03375762	Spain	Aug. 2019-present		Within 8 h, prehospital setting single treatment	Ongoing	—

## Abbreviations

3-MA: 3-Methyladenine
AP1: Activator protein 1
Apaf-1: Apoptotic protease activating factor-1
ATF6: Activating transcription factor 6
Bak: Bcl-2 homologous antagonist killer
Bax: Bcl-2-associated X protein
BBB: Blood-brain barrier
CREB: cAMP-response element binding protein
CHOP: C/EBP homologous protein
Drp1: Dynamin-1-like protein
ER: Endoplasmic reticulum
ERMP1: Endoplasmic reticulum metallopeptidase 1
G-CSF: Granulocyte colony-stimulating factor
GPX: Glutathione peroxidase
GRP78: Glucose-related protein 78
IRE1*α*: Inositol-requiring kinase 1*α*
IRI: Ischemic reperfusion injury
IS: Ischemic stroke
JAK2: Janus kinase 2
JMJD: Jumonji domain-containing proteins
JNK: Jun N-terminal kinase
LPO: Lipid peroxidation
MAPK: Mitogen-activated protein kinase
MCAO: Middle cerebral artery occlusion
NADH: Nicotinamide adenine dinucleotide
NF-*κ*B: Nuclear factor *κ*B
NMDA: N-methyl-D-aspartate
NOS3: Nitric oxide synthase 3
NOXs: NADPH oxidase
OS: Oxidative stress
PARP-1: Poly [ADP-ribose] polymerase 1
PERK: Protein kinase RNA-like endoplasmic reticulum kinase
PI3K: Phosphatidylinositol 3-kinases
PKA: Protein kinase A
POM: Polyoxometalate
RNS: Reactive nitrogen species
ROS: Reactive oxygen species
STAT3: Signal transducer and activator of transcription 3
SOD: Superoxide dismutase
TLR: Toll-like receptors
t-PA: Tissue plasminogen activator
UPR: Unfolded protein response.

## References

[1] Mendelson S. J., Prabhakaran S. (2021). Diagnosis and management of transient ischemic attack and acute ischemic Stroke. *JAMA*.

[2] Mattson M. P., LaFerla F. M., Chan S. L., Leissring M. A., Shepel P. N., Geiger J. D. (2000). Calcium signaling in the ER: its role in neuronal plasticity and neurodegenerative disorders. *Trends in Neurosciences*.

[3] Hetz C. (2012). The unfolded protein response: controlling cell fate decisions under ER stress and beyond. *Nature Reviews. Molecular Cell Biology*.

[4] Sies H. (2015). Oxidative stress: a concept in redox biology and medicine. *Redox Biology*.

[5] Chen H., Yoshioka H., Kim G. S. (2011). Oxidative stress in ischemic brain damage: mechanisms of cell death and potential molecular targets for neuroprotection. *Antioxidants & Redox Signaling*.

[6] Kahl A., Stepanova A., Konrad C. (2018). Critical role of flavin and glutathione in complex I-mediated bioenergetic failure in brain ischemia/reperfusion injury. *Stroke*.

[7] Xue B., Huang J., Ma B., Yang B., Chang D., Liu J. (2019). Astragaloside IV protects primary cerebral cortical neurons from oxygen and glucose deprivation/reoxygenation by activating the PKA/CREB pathway. *Neuroscience*.

[8] Jung J. E., Karatas H., Liu Y. (2015). STAT-dependent upregulation of 12/15-lipoxygenase contributes to neuronal injury after stroke. *Journal of Cerebral Blood Flow and Metabolism*.

[9] Pallast S., Arai K., Pekcec A. (2010). Increased nuclear apoptosis-inducing factor after transient focal ischemia: a 12/15-lipoxygenase-dependent organelle damage pathway. *Journal of Cerebral Blood Flow and Metabolism*.

[10] Yoshida H., Kong Y. Y., Yoshida R. (1998). Apaf1 is required for mitochondrial pathways of apoptosis and brain development. *Cell*.

[11] Sairanen T., Szepesi R., Karjalainen-Lindsberg M. L., Saksi J., Paetau A., Lindsberg P. J. (2009). Neuronal caspase-3 and PARP-1 correlate differentially with apoptosis and necrosis in ischemic human stroke. *Acta Neuropathologica*.

[12] Zhang H., Wang J., Huang J. (2018). Inhibiting Jumoji domain containing protein 3 (JMJD3) prevent neuronal apoptosis from stroke. *Experimental Neurology*.

[13] Wan H., Wang Q., Chen X. (2020). WDR45 contributes to neurodegeneration through regulation of ER homeostasis and neuronal death. *Autophagy*.

[14] Pan C., Prentice H., Price A. L., Wu J. Y. (2012). Beneficial effect of taurine on hypoxia- and glutamate-induced endoplasmic reticulum stress pathways in primary neuronal culture. *Amino Acids*.

[15] Muñoz J. P., Ivanova S., Sánchez‐Wandelmer J. (2013). Mfn2 modulates the UPR and mitochondrial function via repression of PERK. *The EMBO Journal*.

[16] Ogawa S., Kitao Y., Hori O. (2007). Ischemia-induced neuronal cell death and stress response. *Antioxidants & Redox Signaling*.

[17] Mo Y., Sun Y. Y., Liu K. Y. (2020). Autophagy and inflammation in ischemic stroke. *Neural Regeneration Research*.

[18] Han Y., Yuan M., Guo Y. S., Shen X. Y., Gao Z. K., Bi X. (2021). Mechanism of endoplasmic reticulum stress in cerebral ischemia. *Frontiers in Cellular Neuroscience*.

[19] Yao Y., Zhang Y., Liao X., Yang R., Lei Y., Luo J. (2020). Potential therapies for cerebral edema after ischemic stroke: a mini review. *Frontiers in Aging Neuroscience*.

[20] Powers W. J., Rabinstein A. A., Ackerson T. (2018). 2018 guidelines for the early management of patients with acute ischemic stroke: a guideline for healthcare professionals from the American Heart Association/American Stroke Association. *Stroke*.

[21] Prabhakaran S., Ruff I., Bernstein R. A. (2015). Acute stroke Intervention. *JAMA*.

[22] Yaghi S., Willey J. Z., Cucchiara B. (2017). Treatment and outcome of hemorrhagic transformation after intravenous alteplase in acute ischemic stroke: a scientific statement for healthcare professionals from the American Heart Association/American Stroke Association. *Stroke*.

[23] Cid-Castro C., Hernandez-Espinosa D. R., Moran J. (2018). ROS as regulators of mitochondrial dynamics in neurons. *Cellular and Molecular Neurobiology*.

[24] Cho M. H., Kim D. H., Choi J. E., Chang E. J., Seung-YongYoon (2012). Increased phosphorylation of dynamin-related protein 1 and mitochondrial fission in okadaic acid-treated neurons. *Brain Research*.

[25] Zhou L., Zhang Q., Zhang P. (2017). C-Abl-mediated Drp1 phosphorylation promotes oxidative stress-induced mitochondrial fragmentation and neuronal cell death. *Cell Death & Disease*.

[26] Zhang Y., Sun R., Li X., Fang W. (2020). Porcine circovirus 2 induction of ROS is responsible for mitophagy in PK-15 cells via activation of Drp1 phosphorylation. *Viruses*.

[27] Youle R. J., van der Bliek A. M. (2012). Mitochondrial fission, fusion, and stress. *Science*.

[28] Zhao Y. X., Cui M., Chen S. F., Dong Q., Liu X. Y. (2014). Amelioration of ischemic mitochondrial injury and Bax-dependent outer membrane permeabilization by Mdivi-1. *CNS Neuroscience & Therapeutics*.

[29] Zhou K., Chen J., Wu J. (2019). Atractylenolide III ameliorates cerebral ischemic injury and neuroinflammation associated with inhibiting JAK2/STAT3/Drp1-dependent mitochondrial fission in microglia. *Phytomedicine*.

[30] Wei N., Pu Y., Yang Z., Pan Y., Liu L. (2019). Therapeutic effects of melatonin on cerebral ischemia reperfusion injury: role of yap-OPA1 signaling pathway and mitochondrial fusion. *Biomedicine & Pharmacotherapy*.

[31] Chimeh U., Zimmerman M. A., Gilyazova N., Li P. A. (2018). B355252, a novel small molecule, confers neuroprotection against cobalt chloride toxicity in mouse hippocampal cells through altering mitochondrial dynamics and limiting autophagy induction. *International Journal of Medical Sciences*.

[32] Kim T., Mehta S. L., Morris-Blanco K. C. (2018). The microRNA miR-7a-5p ameliorates ischemic brain damage by repressing *α*-synuclein. *Science Signaling*.

[33] Modi J., Menzie-Suderam J., Xu H. (2020). Mode of action of granulocyte-colony stimulating factor (G-CSF) as a novel therapy for stroke in a mouse model. *Journal of Biomedical Science*.

[34] Yin Y., Sun G., Li E., Kiselyov K., Sun D. (2017). ER stress and impaired autophagy flux in neuronal degeneration and brain injury. *Ageing Research Reviews*.

[35] Wu Y. T., Tan H. L., Shui G. (2010). Dual role of 3-methyladenine in modulation of autophagy via different temporal patterns of inhibition on class I and III phosphoinositide 3-kinase. *The Journal of Biological Chemistry*.

[36] Sheng R., Liu X. Q., Zhang L. S. (2012). Autophagy regulates endoplasmic reticulum stress in ischemic preconditioning. *Autophagy*.

[37] Fan T., Huang Z., Chen L. (2016). Associations between autophagy, the ubiquitin-proteasome system and endoplasmic reticulum stress in hypoxia-deoxygenation or ischemia-reperfusion. *European Journal of Pharmacology*.

[38] Zhang X., Yuan Y., Jiang L. (2014). Endoplasmic reticulum stress induced by tunicamycin and thapsigargin protects against transient ischemic brain injury: involvement of PARK2-dependent mitophagy. *Autophagy*.

[39] Feng D., Wang B., Wang L. (2017). Pre-ischemia melatonin treatment alleviated acute neuronal injury after ischemic stroke by inhibiting endoplasmic reticulum stress-dependent autophagy via PERK and IRE1 signalings. *Journal of Pineal Research*.

[40] Hadley G., Neuhaus A. A., Couch Y. (2018). The role of the endoplasmic reticulum stress response following cerebral ischemia. *International Journal of Stroke*.

[41] Li Y., Zhang Y., Fu H. (2020). Hes1 knockdown exacerbates ischemic stroke following tMCAO by increasing ER stress-dependent apoptosis via the PERK/eIF2*α*/ATF4/CHOP signaling pathway. *Neuroscience Bulletin*.

[42] Wei J., Wu X., Luo P. (2019). Homer1a attenuates endoplasmic reticulum stress-induced mitochondrial stress after ischemic reperfusion injury by inhibiting the PERK pathway. *Frontiers in Cellular Neuroscience*.

[43] Chi L., Jiao D., Nan G., Yuan H., Shen J., Gao Y. (2019). miR-9-5p attenuates ischemic stroke through targeting ERMP1-mediated endoplasmic reticulum stress. *Acta Histochemica*.

[44] Tian Y. S., Zhong D., Liu Q. Q. (2018). Upregulation of miR-216a exerts neuroprotective effects against ischemic injury through negatively regulating JAK2/STAT3-involved apoptosis and inflammatory pathways. *Journal of Neurosurgery*.

[45] Dong Y. F., Chen Z. Z., Zhao Z. (2016). Potential role of microRNA-7 in the anti-neuroinflammation effects of nicorandil in astrocytes induced by oxygen-glucose deprivation. *Journal of Neuroinflammation*.

[46] Maccecchini M. L., Chang M. Y., Pan C., John V., Zetterberg H., Greig N. H. (2012). Posiphen as a candidate drug to lower CSF amyloid precursor protein, amyloid-*β* peptide and *τ* levels: target engagement, tolerability and pharmacokinetics in humans. *Journal of Neurology, Neurosurgery, and Psychiatry*.

[47] Yu S. J., Wu K. J., Bae E. (2020). Post-treatment with Posiphen reduces endoplasmic reticulum stress and neurodegeneration in stroke brain. *iScience*.

[48] Turcato F., Kim P., Barnett A. (2018). Sequential combined treatment of pifithrin-*α* and Posiphen enhances neurogenesis and functional recovery after stroke. *Cell Transplantation*.

[49] Santos-Galdiano M., González-Rodríguez P., Font-Belmonte E. (2021). Celecoxib-dependent neuroprotection in a rat model of transient middle cerebral artery occlusion (tMCAO) involves modifications in unfolded protein response (UPR) and proteasome. *Molecular Neurobiology*.

[50] Nita M., Grzybowski A. (2016). The role of the reactive oxygen species and oxidative stress in the pathomechanism of the age-related ocular diseases and other pathologies of the anterior and posterior eye segments in adults. *Oxidative Medicine and Cellular Longevity*.

[51] Carvalho C., Moreira P. I. (2018). Oxidative stress: a major player in cerebrovascular alterations associated to neurodegenerative events. *Frontiers in Physiology*.

[52] Genovese T., Mazzon E., Paterniti I., Esposito E., Bramanti P., Cuzzocrea S. (2011). Modulation of NADPH oxidase activation in cerebral ischemia/reperfusion injury in rats. *Brain Research*.

[53] De Silva T. M., Miller A. A. (2016). Cerebral small vessel disease: targeting oxidative stress as a novel therapeutic strategy?. *Frontiers in Pharmacology*.

[54] Kim J. Y., Park J., Lee J. E., Yenari M. A. (2017). NOX inhibitors - a promising avenue for ischemic stroke. *Experimental Neurobiology*.

[55] Yamaguchi T., Sano K., Takakura K. (1998). Ebselen in acute ischemic stroke: a placebo-controlled, double-blind clinical trial. *Stroke*.

[56] Mei T., Kim A., Vong L. B. (2019). Encapsulation of tissue plasminogen activator in pH-sensitive self-assembled antioxidant nanoparticles for ischemic stroke treatment - Synergistic effect of thrombolysis and antioxidant. *Biomaterials*.

[57] Li S., Jiang D., Ehlerding E. B. (2019). Intrathecal administration of nanoclusters for protecting neurons against oxidative stress in cerebral ischemia/reperfusion injury. *ACS Nano*.

[58] Mizuma A., Yamashita T., Kono S. (2016). Phase II trial of intravenous low-dose granulocyte colony-stimulating factor in acute ischemic stroke. *Journal of Stroke and Cerebrovascular Diseases*.

[59] Zhao X., Strong R., Piriyawat P., Palusinski R., Grotta J. C., Aronowski J. (2010). Caffeinol at the receptor level: anti-ischemic effect of N-methyl-D-aspartate receptor blockade is potentiated by caffeine. *Stroke*.

[60] Martin-Schild S., Hallevi H., Shaltoni H. (2009). Combined neuroprotective modalities coupled with thrombolysis in acute ischemic stroke: a pilot study of caffeinol and mild hypothermia. *Journal of Stroke and Cerebrovascular Diseases*.

[61] Carbone F., Teixeira P. C., Braunersreuther V., Mach F., Vuilleumier N., Montecucco F. (2015). Pathophysiology and treatments of oxidative injury in ischemic stroke: focus on the phagocytic NADPH oxidase 2. *Antioxidants & Redox Signaling*.

[62] Xu J., Wang A., Meng X. (2021). Edaravone dexborneol versus edaravone alone for the treatment of acute ischemic stroke: a phase III, randomized, double-blind, comparative trial. *Stroke*.

[63] Xu J., Wang Y., Wang A. (2019). Safety and efficacy of edaravone dexborneol versus edaravone for patients with acute ischaemic stroke: a phase II, multicentre, randomised, double-blind, multiple-dose, active-controlled clinical trial. *Stroke and Vascular Neurology*.

[64] DeGregorio-Rocasolano N., Martí-Sistac O., Ponce J. (2018). Iron-loaded transferrin (Tf) is detrimental whereas iron-free Tf confers protection against brain ischemia by modifying blood Tf saturation and subsequent neuronal damage. *Redox Biology*.

[65] Abdul Y., Li W., Ward R. (2021). Deferoxamine treatment prevents post-stroke vasoregression and neurovascular unit remodeling leading to improved functional outcomes in type 2 male diabetic rats: role of endothelial ferroptosis. *Translational Stroke Research*.

[66] Millán M., DeGregorio-Rocasolano N., Pérez de la Ossa N. (2021). Targeting pro-oxidant iron with deferoxamine as a treatment for ischemic stroke: safety and optimal dose selection in a randomized clinical trial. *Antioxidants*.

[67] Kim Y., Kim Y. S., Kim H. Y. (2018). Early treatment with poly(ADP-ribose) polymerase-1 inhibitor (JPI-289) reduces infarct volume and improves long-term behavior in an animal model of ischemic stroke. *Molecular Neurobiology*.

[68] Gwag B. J., Lee Y. A., Ko S. Y. (2007). Marked prevention of ischemic brain injury by Neu2000, an NMDA antagonist and antioxidant derived from aspirin and sulfasalazine. *Journal of Cerebral Blood Flow and Metabolism*.

[69] Mondal N. K., Behera J., Kelly K. E., George A. K., Tyagi P. K., Tyagi N. (2019). Tetrahydrocurcumin epigenetically mitigates mitochondrial dysfunction in brain vasculature during ischemic stroke. *Neurochemistry International*.

[70] Stamatovic S. M., Phillips C. M., Martinez-Revollar G., Keep R. F., Andjelkovic A. V. (2019). Involvement of epigenetic mechanisms and non-coding RNAs in blood-brain barrier and neurovascular unit injury and recovery after stroke. *Frontiers in Neuroscience*.

[71] Kalani A., Kamat P. K., Tyagi S. C., Tyagi N. (2013). Synergy of homocysteine, microRNA, and epigenetics: a novel therapeutic approach for stroke. *Molecular Neurobiology*.

[72] Soriano-Tárraga C., Giralt-Steinhauer E., Mola-Caminal M. (2018). Biological age is a predictor of mortality in ischemic stroke. *Scientific Reports*.

[73] Shen Y., Peng C., Bai Q. (2019). Epigenome-wide association study indicates hypomethylation ofMTRNR2L8in large-artery atherosclerosis stroke. *Stroke*.

[74] (1995). tissue plasminogen activator for acute ischemic stroke. *The New England Journal of Medicine*.

[75] Basalay M. V., Wiart M., Chauveau F. (2020). Neuroprotection by remote ischemic conditioning in the setting of acute ischemic stroke: a preclinical two-centre study. *Scientific Reports*.

